# The Book World of Medicine and Science

**Published:** 1898-11-05

**Authors:** 


### THE BOOK WORLD OF MEDICINE AND SCIENCE.

Elements of Pharmacy, Materia Medica, and Therapeutics. By William Whitla, M.A., M.D. Seventh Edition. (London: Henry Renshaw. 1898. Price 10s. 6d.)

Few books have better deserved the continued popularity which they have obtained than Whitla's "Materia Medica." Edition after edition appears, always presenting the same general form, but always, in its internal arrangement, giving evidence of careful and thoughtful supervision, and of a constant endeavour to keep it abreast with the times and to make it useful as a student's manual. It is, indeed, as a student's manual, not limiting its scope either to materia medica, pharmacy, or therapeutics, but endeavouring always to embrace what the student wants to know, that the book has attained its present position, and certainly this edition is quite equal to its predecessors in all these characteristics. The portion of the book which is devoted to a description of the processes of pharmacy is very good, and is likely to be extremely useful to that yearly increasing number of students whose practical experience of the trivial details of pill making and powder folding is but small. Again, the chapter on prescription writing is likely to be of much service to those who have allowed themselves to drift into the lazy way of ordering hospital stock mixtures instead of writing out each prescription in full, and the several pages of Latin words and phrases may prove of service to some. The section which deals with materia medica goes methodically through all the official remedies, giving in each case the composition, the action, and the preparations of the several drugs. Then come nearly two hundred pages on therapeutics and a chapter on non-official remedies. Finally, a list of the formulæ prepared by the British Pharmaceutical Conference and an index of poisons and their antidotes are given. The book is essentially a practical one. This is especially manifest in the earlier chapters; but the same is to be seen in the part devoted to therapeutics, particular attention being paid to the action of the various drugs in the treatment of disease rather than to their mere physiological effects. A few little errors are to be found here and there which we think ought hardly to occur in a book which is in its seventh edition. For instance, it is stated that 20 grains of citric acid in an effervescing mixture will saturate 41 grains of carbonate of sodium or 24 grains of bicarbonate of sodium. Surely a transposition is wanted here. Again, the liquor morphinæ tartratis is said to be prepared by dissolving morphine tartrate 17½ grains in distilled water and alcohol (90 per cent.), of each 1 oz., and making up to 4 ozs. with distilled water. But it is a pity, both here and all through the book, that we are not told when fluid ounces are intended. Still the book is likely to be very useful, and certainly it contains a great deal of information in a very convenient form.

A Synopsis of Surgery. By R. F. Tobin, F.R.C.S.I., Surgeon to St. Vincent's Hospital, Dublin. (London: J. and A. Churchill; Dublin: Fannin and Co. 1898. Price 6s. 6d.)

This is an enlarged note book on surgery, printed on thin paper, and interleaved, so that the student can fill it out as he thinks fit. We venture, however, to doubt whether the student will do anything of the sort. A book so nicely printed and nicely bound, with gilt edges and rounded corners, seems hardly fitted for rough scribbling, nor indeed does the planning of the contents appear such as to make the book very useful as a groundwork for notes. As a short and easily read *résumé* of the main facts of surgery the work is worthy of high praise, and it is quite possible that it may so nearly follow the lines of the lectures delivered by its author that it may serve very well as a note book to his own discourses, but that it is likely to facilitate in any way the taking of notes of any other lectures we do not believe. Nevertheless, as an introduction to surgery, a book in which the student can find in few words the main facts about any class of disease or injury to which his attention may at the moment be directed, the work before us is a very good one.

There is no doubt that the size to which many of the most popular and extensively used works on surgery have attained often leads students to put off referring to them in regard to the points that arise in their daily work until a more convenient season. Little as we like small books on great subjects, it is obvious enough that to the student in the wards it is a great advantage to have actually in his pocket, for immediate reference, a handy book on surgery, and for such a purpose this synopsis seems eminently suitable. It would, however, be more suitable still if it had a good table of contents and a better index. An index to a work on surgery can hardly be spoken of as complete when neither "cancer," "carcinoma," nor "malignant" appears in its columns. It is to be noted that no details are given as to operative treatment, and, indeed, throughout the book treatment is very cursorily dealt with. It is, indeed, essentially a book for the use of students working in the wards. Its aim is so to systematise the clinical experience there gained as to make it the foundation of surgical knowledge. This it does well, and in saying this we feel that we could not be giving it higher praise. But it must not be looked upon as a work of reference for the practitioner.

A Synopsis of the British Pharmacopœia, 1898. Compiled by H. Wippell Gadd. (London: Baillière, Tindall, and Cox. Third Edition. 1898. Price, in paper, 6d.; in cloth, 1s.)

On the first issue of this volume we noticed it favourably as being among the best of the little handbooks which came into existence on the publication of the new Pharmacopœia. It seems probable, however, that it will have a longer life than some of its competitors from the fact that it is not merely an account of changes, but, as its name implies, gives a complete synopsis of the whole of the present edition of the Pharmacopœia. In fact, it is an amplified edition of the very useful index of the latter publication, and thus gives most of the information which the prescribing practitioner requires.

